# Sonographic imaging of the great auricular nerve

**DOI:** 10.1097/MD.0000000000047336

**Published:** 2026-01-23

**Authors:** Mohamed A. Bedewi, Yomna S. Habib, Mamdouh A Kotb, Daifallah Mohamed Almalki, Naif Abdurhman Alrudian, Bader A. Alhariqi, Mohammed Saad Alqahtani, Steven B. Soliman, Kholoud J. Sandougah, Akram Z. Abdelaal, Ramy Abdelnaby

**Affiliations:** a Department of Internal Medicine, College of Medicine, Prince Sattam Bin Abdulaziz University, Al-Kharj, Kingdom of Saudi Arabia; b Department of Radiology, National Cancer Institute, Cairo University, Cairo, Egypt; c Neurology department, Minia University, College of Medicine, Minia, Egypt; d Department of Family and Community Medicine, Prince Sattam Bin Abdulaziz University, College of Medicine, Al-Kharj, Kingdom of Saudi Arabia; e Medical Imaging Administration, Radiology Department, Altakassusi Alliance Medical LLC, Riyadh, Saudi Arabia; f Department of Radiology, Division of Musculoskeletal Radiology, University of Michigan, Ann Arbor, MI; g Department of Radiology, College of Medicine, Imam Mohammad Ibn Saud Islamic University (IMSIU), Riyadh, Kingdom of Saudi Arabia; h Department of Oral and Maxillofacial Surgery, Alahrar Educational Hospital, Zagazig, Egypt; i Department of Geriatrics, St. Joseph Hospital, Prüm, Germany.

**Keywords:** cross-sectional area, great auricular nerve, reference values, ultrasound

## Abstract

The aim of this study is to assess the ability of ultrasound to identify and estimate the normal cross-sectional area (CSA) of the great auricular nerve (GAN) at a defined landmark in healthy adults. The study sample included 80 GANs in 40 participants (11 males, 29 females), mean age 36.5 years, mean height 160.1 cm, mean weight 63 kg, mean body mass index 24.4 kg/m^2^. Two radiologists separately obtained sonographic images of the bilateral GANs. Each participant was scanned 3 times bilaterally to assess for intraobserver reliability. The mean CSA of the bilateral GANs was 0.8 mm^2^ (range 0.4–2.7 mm^2^). The GAN CSA positively correlated with weight, height, and body mass index. This study confirms the ability of ultrasound to reliably assess the GAN at a defined landmark and estimate its normal CSA. Knowledge of how to identify and measure the GAN would be expected to help decrease complications during procedures and help establish normal reference values.

## 1. Introduction

The great auricular nerve (GAN) arises from the ventral cervical rami C2 and C3 spinal roots and is considered the largest superficial ascending sensory branch of the cervical plexus. The GAN emerges along the superficial aspect of the sternocleidomastoid muscle (SCM) and ascends vertically and obliquely across the SCM, dividing into anterior and posterior branches at the postero-inferior aspect of the ear lobe. It innervates the skin over the posterior auricular region, external ear, and the parotid gland.^[1–3]^

The GAN is of special interest for a target in regional anesthesia procedures especially in mastoid and tympanic surgeries.^[4]^ A targeted GAN block is also important in cases of auricular neuralgia. Furthermore, preservation of the GAN in parotid and other neck surgeries is considered a beneficial outcome. However, potential nerve damage of GAN is still a possibility during plastic surgical procedures. The GAN can also be used in facial nerve grafting.^[5,6]^ Knowledge of its normal location and course and how to identify the GAN using ultrasound (US) would be expected to decrease procedural complications related to the GAN. Although GAN neuropathy is rare, it has been reported in leprosy with presence of neuropathic pain.^[7]^ Additionally, enlargement of the GAN has been reported in cases of Charcot-Marie-Tooth with US demonstrating nerve enlargement and thickening.^[8]^ The GAN could also be invaded by both malignant and benign peripheral nerve sheath tumors including schwannomas.^[9]^ This further highlights the importance of how to identify and measure the GAN sonographically. Reference values for the cross-sectional area (CSA) of the peripheral nerves are important for establishment of accurate diagnosis in different neuropathies and traumatic injuries. Only a few studies have been performed evaluating the cranial nerves^[10]^ The aim of this study is to assess the ability of US to identify and estimate the normal CSA of the GAN at a defined landmark in healthy adults.

## 2. Methods

### 2.1. Participants

After institutional review board approval, participants of the study were recruited between October 2022 and October 2023, and written informed consent was obtained. Inclusion criteria included men and women >18 years of age. Subjects with prior nerve injury, neck surgery, and neck scars were excluded from the study. For each participant, data including age, sex, body mass index (BMI), weight, and height were recorded.

### 2.2. Technique

**Figure 1.**
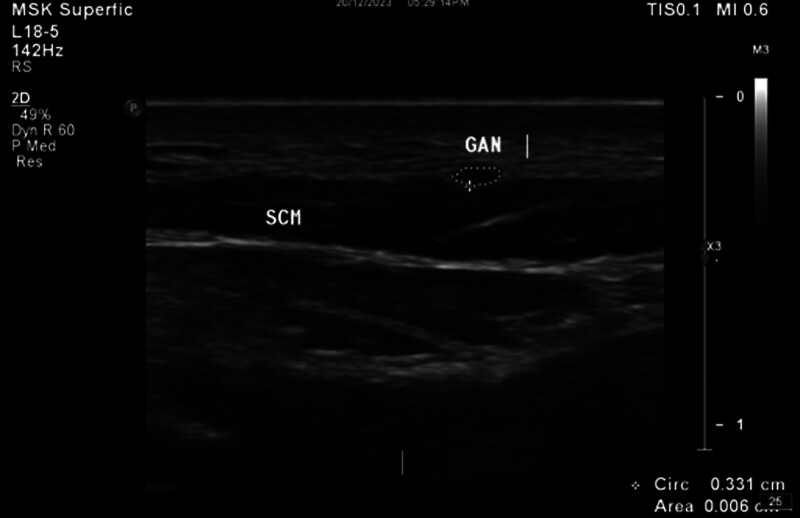
Short axis view of the GAN. CCA = common carotid artery, GAN = great auricular nerve, SCM = sternocleidomastoid muscle.

All subjects were scanned separately by 2 radiologists; 1 (M.B) with 10 years’ experience in neuromuscular US, and the second (Y.S) with 3 years’ experience in neuromuscular US. All studies used an L12–5 MHz linear transducer and occasionally used L18-5 linear transducer (Epic 7 version1.5, Ultrasound system: Philips, Bothell, WA). Each participant was scanned 3 times to assess intraobserver reliability with the US transducer completely removed from the skin between each measurement. In order to image the GAN, all subjects were placed in a supine position with the neck slightly extended. The GAN was identified as an oval/drop-like hypoechoic structure on the deep (dorsal) surface of the SCM near its cranial border. The CSA of the GAN was measured using the tracer method by measuring inside the hyperechoic epineurium (Fig. 1).

### 2.3. Statistical analysis

The mean CSA were compared between both sides using Wilcoxon signed rank test. Paired sample t test was also used to compare the CSA of the right and left GAN. The correlations between the CSA of the scanned nerves, age, weight, height, and BMI were evaluated using Pearson correlation coefficient (r). All data were presented as mean standard deviation (SD) and range. Statistical analysis was performed using Statistical Package for the Social Sciences (SPSS) version 21 software (SPSS Inc, Chicago). A *P*-value of <.05 was considered significant.

## 3. Results

The study sample included 40 participants (11 males, (27.5 %) and, 29 females), mean age 36.5 ± 7.3 (range:26–57), mean height 160.1 cm ± 8.9 (range: 150–180), mean weight 63 kg ± 13.1 (range: 43–102), mean BMI kg/m^2^ 24.4 ± 3.7 (range 16.8–35.3). The intraobserver reliability calculations resulted in an overall intra-class correlation coefficient of 0.80. The mean CSA of the right GAN was 0.8 mm^2^ (range 0.4–1.2 ± 0.2 SD). The mean CSA of the left GAN was 0.9 mm^2^ (range 0.4–2.7 ± 0.5 SD). The mean CSA of the bilateral GANs was 0.8 mm^2^ (range 0.4–2.7 ± 0.4 SD). Table 1 shows the descriptive statistics and CSA measurements of the GAN. Table 2 demonstrates correlations between age, weight, BMI, and height, with the CSA of the GAN. We compared the values of the mean CSA of the right and left GAN CSA (*P* = .075) in each subject and no significant statistical differences were noted. The CSA showed positive correlation with weight (*P* = .001), height (*P* = .012), and BMI (*P* = .017). No correlation was found with age. The gender difference regarding the GAN CSA was 0.8 ± 0.3 mm^2^ in women and 1 ± 0.6 mm^2^ in men, albeit not statistically significant (*P* = .083).

**Table 1:** Demographic characteristics of study participants and CSA measurements (mean ± standard deviation).

	N	Minimum	Maximum	Mean	Std. deviation
Age	40	26	57	36.5	7.3
Weight in kg	40	43	102	63.0	13.1
Height in cm	40	150	180	160.1	8.9
BMI	40	16.8	35.3	24.4	3.7
CSA right mm^2^	40	0.4	1.2	0.8	0.2
CSA left mm^2^	40	0.4	2.7	0.9	0.5
CSA both sides mm^2^	80	0.4	2.7	0.8	0.4

BMI = body mass index, CSA = cross-sectional area.

**Table 2:** Demonstrates the correlations between age, weight, BMI, and height, with the CSA of GAN.

		Right and Left sides	LT CSA	RT CSA
Age	Pearson correlation	−.003	−.053	.104
	Sig. (2-tailed)	.982	.743	.521
	N	80	40	40
Weight in kg	Pearson correlation	.359**	.432**	.292
	Sig. (2-tailed)	0.001	.005	.067
	N	80	40	40
Height in cm	Pearson correlation	.279*	.388*	.115
	Sig. (2-tailed)	0.012	.013	.480
	N	80	40	40
BMI	Pearson correlation	.266*	.281	.297
	Sig. (2-tailed)	0.017	.079	.063
	N	80	40	40

BMI = body mass index, CSA = cross-sectional area, GAN = great auricular nerve.

* . Correlation is significant at the .05 level (2-tailed).

** . Correlation is significant at the .01 level (2-tailed).

## 4. Discussion

The present study demonstrates reliable identification of the GAN on US imaging.

The SCM was a clear anatomical landmark for identification of the GAN, with the nerve identified as oval or drop-like structure on the superficial surface of the SCM. Both static imaging and a sweeping method of identification of the nerve are mentioned in the literature. We believe that the sweeping method, in which the transducer is moved in a cranial to caudal direction and back and forth, is most useful in identifying small nerves of the neck like the spinal accessory nerve and the long thoracic nerve. In our study, static exam was first performed, and if the nerve was not clear, we resorted to the sweeping method.^[11]^

Limited data was found in the literature regarding estimating the CSA of the GAN using US. The mean CSA of the GAN in our study was 0.8 mm^2^. Our results were comparable with available research, Curcean et al (0.90 mm^2^ ± 0.53 on the left and 0.79 mm^2^ ± 0.71 on the right).^[12]^ and Christ et al (1 mm) diameter.^[13]^ Samal et al reported higher values (0.14 cm ± 0.03, range 0.08–0.2), however, this group performed the study on a smaller sample size (10 healthy volunteers) as compared to our study (40 healthy volunteers). This difference in size could be attributed to differences in the site of measurement and different study cohorts. Positive correlation was found between the GAN CSA and weight, and BMI coinciding with Curcean et al Additionally, our study showed positive correlation between CSA and height. There was no statistical difference between males and females or between right and left sides coinciding with Curcean et al This study has several limitations. First is the small sample size although we chose a sample size that would show statistical significance and that was larger than prior studies. Second, our study was only confined to healthy volunteers and did not include pathological conditions. This was in order to compare with CSA measurements of the normal GAN. Future larger studies should evaluate the CSA of the GAN in pathologic conditions. In conclusion, this study confirms the ability of US to reliably to assess the GAN at a defined landmark and estimate its CSA in healthy subjects. Knowledge of how to identify and measure the GAN during procedures would be expected to decrease the risk of associated complications and help establish normal reference values.

## Acknowledgments

The authors thank the Deanship of Scientific Research at Prince Sattam bin Abdulaziz University.

## Author contributions

**Conceptualization:** Mohamed A. Bedewi, Yomna S. Habib, Mamdouh A Kotb, Daifallah Mohamed Almalki, Naif Abdurhman Alrudian, Mohammed Saad Alqahtani, Steven B. Soliman, Kholoud J. Sandougah.

**Data curation:** Mohamed A. Bedewi, Yomna S. Habib, Mamdouh A Kotb.

**Formal analysis:** Mamdouh A Kotb.

**Investigation:** Mohamed A. Bedewi, Yomna S. Habib, Naif Abdurhman Alrudian, Bader A. Alhariqi, Kholoud J. Sandougah, Ramy Abdelnaby.

**Methodology:** Mohamed A. Bedewi, Yomna S. Habib, Daifallah Mohamed Almalki, Naif Abdurhman Alrudian, Bader A. Alhariqi.

**Project administration:** Mohamed A. Bedewi, Daifallah Mohamed Almalki, Bader A. Alhariqi, Mohammed Saad Alqahtani, Steven B. Soliman, Kholoud J. Sandougah, Akram Z. Abdelaal, Ramy Abdelnaby.

**Supervision:** Mohamed A. Bedewi, Yomna S. Habib, Mohammed Saad Alqahtani, Kholoud J. Sandougah, Akram Z. Abdelaal, Ramy Abdelnaby.

**Validation:** Mamdouh A Kotb.

**Visualization:** Mohamed A. Bedewi.

**Writing – original draft:** Mohamed A. Bedewi.

**Writing – review & editing:** Mohamed A. Bedewi, Steven B. Soliman.
